# The use of kDNA minicircle subclass relative abundance to differentiate between *Leishmania* (*L.*) *infantum* and *Leishmania* (*L.*) *amazonensis*

**DOI:** 10.1186/s13071-017-2181-x

**Published:** 2017-05-16

**Authors:** Marcello Ceccarelli, Luca Galluzzi, Aurora Diotallevi, Francesca Andreoni, Hailie Fowler, Christine Petersen, Fabrizio Vitale, Mauro Magnani

**Affiliations:** 1 Department of Biomolecular Sciences, University of Urbino “Carlo Bo”, Urbino, PU Italy; 2 Department of Epidemiology, College of Public Health, University of Iowa, Iowa City, IA USA; 3 Istituto Zooprofilattico Sperimentale of Sicily “A Mirri”, Palermo, PA Italy

**Keywords:** *Leishmania* (*L*.) *infantum*, *Leishmania* (*L*.) *amazonensis*, qPCR, HRM, kDNA, Minicircles

## Abstract

**Background:**

Leishmaniasis is a neglected disease caused by many *Leishmania* species, belonging to subgenera *Leishmania* (*Leishmania*) and *Leishmania* (*Viannia*)*.* Several qPCR-based molecular diagnostic approaches have been reported for detection and quantification of *Leishmania* species. Many of these approaches use the kinetoplast DNA (kDNA) minicircles as the target sequence. These assays had potential cross-species amplification, due to sequence similarity between *Leishmania* species. Previous works demonstrated discrimination between *L.* (*Leishmania*) and *L.* (*Viannia*) by SYBR green-based qPCR assays designed on kDNA, followed by melting or high-resolution melt (HRM) analysis. Importantly, these approaches cannot fully distinguish *L.* (*L*.) *infantum* from *L.* (*L*.) *amazonensis*, which can coexist in the same geographical area.

**Methods:**

DNA from 18 strains/isolates of *L.* (*L*.) *infantum*, *L.* (*L*.) *amazonensis*, *L.* (*V.*) *braziliensis*, *L.* (*V.*) *panamensis*, *L.* (*V.*) *guyanensis*, and 62 clinical samples from *L.* (*L*.) *infantum*-infected dogs were amplified by a previously developed qPCR (qPCR-ML) and subjected to HRM analysis; selected PCR products were sequenced using an ABI PRISM 310 Genetic Analyzer. Based on the obtained sequences, a new SYBR-green qPCR assay (qPCR-ama) intended to amplify a minicircle subclass more abundant in *L.* (*L*.) *amazonensis* was designed.

**Results:**

The qPCR-ML followed by HRM analysis did not allow discrimination between *L.* (*L*.) *amazonensis* and *L.* (*L*.) *infantum* in 53.4% of cases. Hence, the novel SYBR green-based qPCR (qPCR-ama) has been tested. This assay achieved a detection limit of 0.1 pg of parasite DNA in samples spiked with host DNA and did not show cross amplification with *Trypanosoma cruzi* or host DNA. Although the qPCR-ama also amplified *L.* (*L*.) *infantum* strains, the C_q_ values were dramatically increased compared to qPCR-ML. Therefore, the combined analysis of C_q_ values from qPCR-ML and qPCR-ama allowed to distinguish *L.* (*L*.) *infantum* and *L.* (*L*.) *amazonensis* in 100% of tested samples.

**Conclusions:**

A new and affordable SYBR-green qPCR-based approach to distinguish between *L.* (*L*.) *infantum* and *L.* (*L*.) *amazonensis* was developed exploiting the major abundance of a minicircle sequence rather than targeting a hypothetical species-specific sequence. The fast and accurate discrimination between these species can be useful to provide adequate prognosis and treatment.

**Electronic supplementary material:**

The online version of this article (doi:10.1186/s13071-017-2181-x) contains supplementary material, which is available to authorized users.

## Background

Leishmaniasis is a spectral neglected disease caused by many *Leishmania* species, primarily transmitted by phlebotomine sand flies [1]. Leishmaniasis is a public health problem in 98 countries, in both rural and urban areas. About 12 million people are currently affected by the disease; in particular, 0.2–0.4 million cases per year of visceral leishmaniasis (VL) and 0.7–1.2 million cases per year of cutaneous leishmaniasis (CL) have been estimated [2]. *Leishmania* parasites are classified in three different subgenera: *Leishmania* (*Leishmania*), *Leishmania* (*Viannia*) and *Leishmania* (*Sauroleishmania*). Parasites in the subgenera *Leishmania* and *Viannia* infect mammals, whereas the *Sauroleishmania* infect reptiles [3]. Over 30 *Leishmania* species have been identified, including many that are non-pathogenic or of minor medical importance. The *Leishmania donovani* complex belongs to the subgenus *Leishmania* (*Leishmania*), the etiological agent of VL and CL, and includes *L.* (*L*.) *infantum* and *L.* (*L*.) *donovani*. The species belonging to the subgenus *Leishmania* (*Viannia*) are etiological agents of CL and mucocutaneous leishmaniasis.

An accurate diagnostic method that allows the distinction between *Leishmania* species with overlapping geographical distributions is needed [4]. Since isoenzymatic characterization is laborious and time-expensive, various molecular approaches have been developed based on detection of different *Leishmania* target sequences [5, 6]. In particular, the conserved region of *Leishmania* kinetoplast DNA (kDNA) minicircles has been used as a specific target for real-time PCR assays, able to distinguish Old World [7, 8] and New World species [9]. The kDNA is situated at the base of the flagellum and contains thousands of minicircles and dozens of maxicircles concatenated in a giant network [10]. The high number of minicircles (10,000–26,000 copies/parasite) [11–13] make them an attractive target for *Leishmania* detection with high sensitivity [14]. However, the minicircle network is composed of different minicircle subclasses conserved in different *Leishmania* species [15, 16]. Therefore, most of the available PCR or qPCR assays designed for a single species can potentially amplify more than one *Leishmania* species [13, 14, 17].

Previous works demonstrated the possibility to discriminate between *L.* (*Leishmania*) and *L.* (*Viannia*) by SYBR green-based qPCR assays designed on kDNA conserved sequences, followed by melting or high-resolution melt (HRM) analysis [13, 18]. These approaches did not efficiently distinguish *L.* (*L.*) *infantum* from *L.* (*L*.) *amazonensis*, which can coexist in the same geographic area. These species infect both humans and dogs [19], and their differentiation is critical for correct treatment. In fact, both species can present with cutaneous manifestations, but *L.* (*L*.) *amazonensis* can also cause diffuse disease that does not respond well to current treatments [5]. Moreover, the use of immunosuppressant may alter the clinical course of Leishmaniasis (e.g. viscerotropic strains can result in cutaneous lesions, and dermotropic strains can result in visceral disease). These issues are particularly important in South America, where both species are endemic, and deforestation and uncontrolled urbanisation are responsible for the growing incidence of leishmaniasis [20].

The aim of this work is to investigate the kDNA sequence variability among different *Leishmania* species and to exploit these differences to develop a qPCR-based analysis that differentiates *L.* (*L*.) *infantum* from *L.* (*L*.) *amazonensis*.

## Methods

### *Leishmania* strains, clinical samples and DNA extraction

The DNA from promastigotes of *Leishmania* spp*.* isolates listed in Table 1 was chelex-purified. Conjunctival swabs (CS) or buffy coat (BC) samples from 21 dogs diagnosed with leishmaniasis were provided by the veterinary clinic “Santa Teresa” (Fano, Italy). The DNA from CS and BC samples was extracted as previously described [21]. The *L. amazonensis* and *L.* (*Viannia*) spp. clinical isolates were provided by Instituto de Patología Experimental (IPE), Facultad de Ciencias de la Salud - Universidad Nacional de Salta- Argentina and typed at the species level at the Institute of Biomedicine and Molecular Immunology, CNR (Palermo, Italy) [13]. The *L. infantum* clinical isolates have been characterised by multilocus enzyme electrophoresis (MLEE) at Istituto Superiore di Sanità (Rome, Italy).

**Table 1:** *Leishmania* strains/isolates used in this study

Species	Strain or isolate	Zymodeme
*L.* (*L.*) *infantum*	MHOM/TN/80/IPT1	MON-1
*L.* (*L.*) *infantum*	MHOM/FR/78/LEM75	MON-1
*L.* (*L.*) *infantum*	MHOM/ES/81/BCN1	MON-29
*L.* (*L.*) *infantum*	MHOM/DZ/82/LIPA59	MON-24
*L.* (*L.*) *infantum*	MHOM/IT/93/ISS822	MON-201
*L.* (*L.*) *infantum*	MHOM/IT/86/ISS218	MON-72
*L.* (*L.*) *infantum*	Clinical isolate V2921	MON-1
*L.* (*L.*) *infantum*	Clinical isolate 31u	MON-1
*L.* (*L.*) *infantum*	Clinical isolate 49u	MON-1
*L.* (*L.*) *infantum*	Clinical isolate 791	MON-1
*L.* (*L.*) *infantum*	Clinical isolate 10816	MON-1
*L.* (*L.*) *amazonensis*	MHOM/BR/00/LTB0016	na
*L.* (*L.*) *amazonensis*	IFLA/BR/67/PH8	MON-41
*L.* (*L.*) *amazonensis*	Clinical isolate	na
*L.* (*V.*) *panamensis*	Clinical isolate	na
*L.* (*V.*) *guyanensis*	Clinical isolate	na
*L.* (*V.*) *braziliensis*	Clinical isolate	na
*L.* (*V.*) *braziliensis*	MHOM/BR/75/M2904	na

*Abbreviation*: *na* not applicable

The DNA extracted from isolates and strains was quantified using a Qubit fluorometer (Life Technologies, Carlsbad, USA). Human and canine DNA from uninfected donors, purified using the DNeasy Blood & Tissue kit (Qiagen, Valencia, USA), as well as DNA from *Trypanosoma cruzi* were used as negative controls.

### qPCR assays

An 111 bp KDNA region was amplified by a qPCR assay using primers MLF and MLR (referred as qPCR-ML from hereon) as previously described [13]. Briefly, PCR reactions were carried out in 25 μl volume with 1 μl template DNA (corresponding to 0.1–1 ng parasite DNA) and 24 μl SYBR green PCR master mix (Diatheva srl, Fano, Italy) containing 1 U *Taq* Polymerase and 200 nM of each primer in a Rotor-Gene 6000 instrument (Corbett Life Science, Mortlake, Australia). The amplification conditions were: 94 °C for 10 min, 40 cycles at 94 °C for 30 s, 60 °C for 20 s and 72 °C for 20 s. At the end of each run, a melting curve analysis was performed from 78 to 92 °C, with a slope of 1 °C/s, and 5 s at each temperature. The reactions were performed in duplicate or triplicate.

**Fig. 1:**
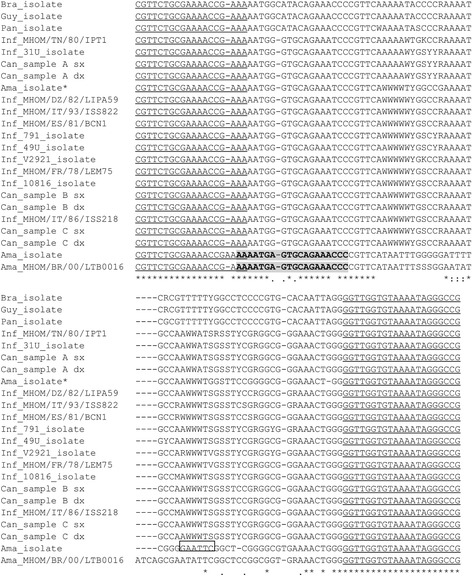
CLUSTAL multiple alignments of qPCR-ML amplicon sequences performed by MUSCLE. The MLF and MLR primer sequences are *underlined*; the sequence of LMi-amaF primer is in bold. The *EcoR*I restriction site in *L.* (*L.*) *amazonensis* isolate is boxed. *Sequence of non-digested products after *EcoR*I restriction

To amplify *L.* (*L.*) *amazonensis* DNA minicircles, a new qPCR assay (referred as qPCR-ama from hereon) was designed using the forward primer LMi-amaF (5′-AAA ATG AGT GCA GAA ACC C-3′) with the reverse primer MLR. The LMi-amaF primer was designed specifically for this work exploiting two mismatches (G/A and T/C) among *L.* (*L.*) *infantum* and *L.* (*L.*) *amazonensis* sequences (Fig. 1). The LMi-amaF sequence was tested with BLAST against the non-redundant nucleotide database (organism limited to *Leishmania*). The position of all primers in the qPCR-ML amplicons is shown in Fig. 1. The qPCR-ama was performed at the same conditions described above. A standard curve for qPCR-ama was established using *L.* (*L.*) *amazonensis* MHOM/BR/00/LTB0016 DNA serial dilutions, ranging from 1.0 to 1 × 10^-4^ ng. The standard curve concentration was expressed as ng/μl. To evaluate the potential interference of host DNA as background in the qPCR analysis, 30 ng of human DNA were spiked in the reaction tubes. The cycle threshold (C_q_) values were determined using the quantitation analysis of the Rotor-Gene 6000 software, setting a threshold to 0.15.

### High-resolution melt (HRM) analysis

The qPCR-ML amplicons, obtained by all stains/isolates listed in Table 1 and by 62 samples from 21 dogs affected by leishmaniasis, were analysed in duplicate by HRM protocol on a Rotor-Gene 6000 instrument as described previously [13] with slight modifications. Briefly, HRM was carried out over the range from 78 °C to 92 °C, rising at 0.1 °C/s and waiting for 2 s at each temperature. Each sample was run in duplicate or triplicate, and the gain was optimised before melting on all tubes. HRM curve analysis was performed with the derivative of the raw data, after smoothing, with the Rotor-Gene 6000 software. Only samples with C_q_ values ˂ 30 were considered for subsequent analysis [13, 22].

### PCR product sequencing

The qPCR-ML products obtained from the canine clinical samples and the strains/isolates (Table 1, except for IFLA/BR/67/PH8 and MHOM/BR/75/M2904 strains), were purified using the MinElute PCR purification kit (Qiagen) and directly sequenced using both MLF and MLR primers. DNA sequencing was performed using the BigDye Terminator v. 1.1 Cycle Sequencing Kit on ABI PRISM 310 Genetic Analyzer (Applied Biosystems, Foster City, USA). Sequences were manually edited and aligned using default options in MUSCLE [23]. Phylogenetic and nucleotide composition analyses were conducted using MEGA version 6 [24].

### RFLP analysis

The RFLP analysis was performed on qPCR-ML products with *EcoR*I restriction enzyme (Roche Life Sciences, Indianapolis, USA), following the manufacturer’s protocol. The *EcoR*I digestion was performed at 37 °C for 2 h in a 20 μl mixture containing 7 U of enzyme and 50–100 ng of PCR products. The digestion mixtures were analysed on a 3% high-resolution MetaPhor (Cambrex, East Rutherford, USA) agarose gel, and the selected products were excised, purified with MinElute PCR purification kit (Qiagen) and sequenced as described above.

### Statistical analysis

Statistical analysis was performed with GraphPad InStat version 3.06 (GraphPad Software, San Diego, CA). Differences among T_m_ values were evaluated using an Unpaired t-test with Welch’s correction. Normality distribution was assessed by D’Agostino & Pearson omnibus normality test (alpha = 0.05).

## Results

### Inter- and intra-specific genetic variability of kDNA minicircles

**Fig. 2:**
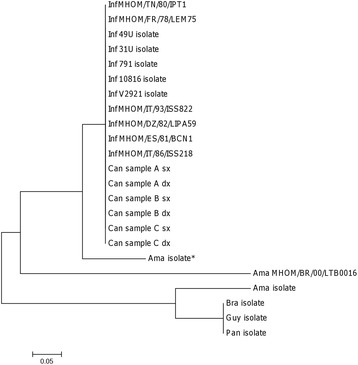
Maximum likelihood phylogenetic tree of qPCR-ML amplicons. The tree was constructed with MEGA 6 software using Tamura-Nei model. *Sequence of non-digested product after *EcoR*I restriction

To investigate the genetic variability in the kDNA minicircle sequences amplified by qPCR-ML assay, the PCR products from all strains/isolates reported in Table 1 (except *L. amazonensis* IFLA/BR/67/PH8 and *L. braziliensis* MHOM/BR/75/M2904) and 6 CS samples from 3 dogs with leishmaniasis were directly and bidirectionally sequenced. The electropherograms showed superimposed peaks in some region, suggesting a further kDNA sequence heterogeneity. Therefore, sequences were manually edited before alignment. Numerous polymorphic loci were found (Fig. 1). The *Leishmania* (*Viannia*) subgenus isolates had significantly lower GC content respect to *Leishmania* (*Leishmania*) isolates (47.4% ± 0.2% and 51.3% ± 0.5%, respectively) (Mann–Whitney test, U = 3.00, *P* = 0.015), explaining melting differences found previously [13]. The sequences of *Leishmania* (*Viannia*), *L.* (*L.*) *amazonensis* isolates/strains and *L.* (*L.*) *infantum* strains/clinical samples, clustered separately (Fig. 2).

A restriction site for *EcoR*I was evidenced in the *L.* (*L.*) *amazonensis* isolate, but it was not confirmed in *L.* (*L.*) *amazonensis* MHOM/BR/00/LTB0016 strain (Fig. 1), making it not feasible to develop a PCR-RFLP assay to distinguish *L.* (*L.*) *amazonensis* from *L.* (*L.*) *infantum*. Moreover, the RFLP analysis of the *L.* (*L.*) *amazonensis* isolate revealed a partial digestion of the PCR product (Additional file 1: Figure S1). The non-cleaved band was extracted from the gel and directly sequenced. The lack of a *EcoR*I restriction site, as well as many similarities with the *L.* (*L.*) *infantum* sequences (Figs. 1 and 2), showed the presence of different minicircle classes amplified by the qPCR-ML assay.

### HRM analysis alone does not fully discriminate between *L.* (*L*.) *infantum* and *L.* (*L*.) *amazonensis*

**Fig. 3:**
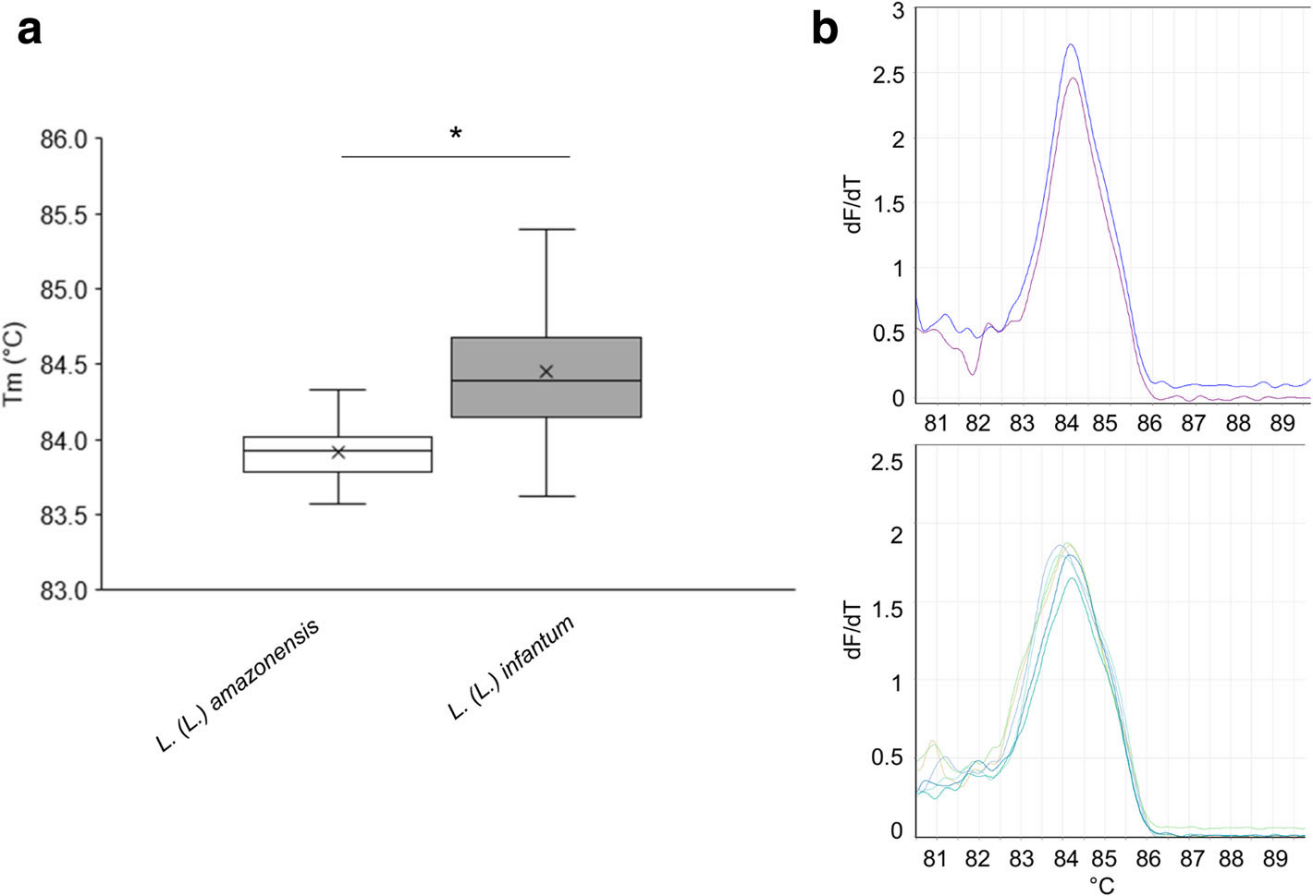
T_m_ values distribution of qPCR-ML amplicons of *L.* (*L.*) *amazonensis* and *L.* (*L.*) *infantum* with single melting peak. **a** Box-and-whisker plot is representing qPCR-ML amplicons T_m_ distribution. The chart shows the distribution of data into quartiles, the smallest and the largest observation, and the mean (x). No outliers were identified. **b** Examples of overlapping melting curves obtained from *L.* (*L.*) *amazonensis* MHOM/BR/00/LTB0016 (T_m_ 84.10–84.15 °C) (upper panel) and *L.* (*L.*) *infantum* MHOM/IT/86/ISS218 (T_m_ 84.15–84.20 °C), clinical isolate 791 (T_m_ 84.10–84.15 °C), clinical isolate 10816 (T_m_ 83.93 °C) (lower panel). These melting curves, showed in duplicates, represent an example of lack of discrimination between *L.* (*L.*) *infantum* and *L.* (*L.*) *amazonensis*

Previously, we demonstrated that HRM analysis of qPCR-ML products can discriminate between the *Leishmania* subgenera *Leishmania* and *Viannia*, and can differentiate the *L.* (*L.*) *infantum* MHOM/TN/80/IPT1 reference strain (two melting peaks) from *L.* (*L.*) *amazonensis* (one melting peak) [13]. However, HRM analysis performed on several clinical specimens from dogs infected by *L.* (*L.*) *infantum*, showed two peaks comparable to *L.* (*L.*) *infantum* MHOM/TN/80/IPT1 or a profile with a single melting peak, suggesting a kDNA minicircle parasite variability [13]. To confirm and further extend these data, we performed HRM analysis on qPCR-ML products obtained by amplification of *L.* (*L.*) *infantum* strains/isolates listed in Table 1 and 62 canine clinical samples from 21 dogs infected by *L.* (*L.*) *infantum*. The results showed that 22% and 56% of the *L.* (*L.*) *infantum* amplicons presented a double melting peak and a single melting peak, respectively. The remaining 22% of samples was undetermined due to lack of reproducibility between replicates. *L.* (*L.*) *amazonensis* amplicons always had a single melting peak. T_m_ values between *L.* (*L.*) *amazonensis* isolate, and MHOM/BR/00/LTB0016 strain was not significantly different (unpaired t-test with Welch’s correction, *t* = 1.23, *P* = 0.246). T_m_ values of strain IFLA/BR/67/PH8 were not considered due to late amplification (C_q_ ˃ 30) and consequent lack of reproducible T_m_ profiles [22]. The mean T_m_ of *L.* (*L.*) *amazonensis* amplicons was significantly different from the mean T_m_ of *L.* (*L.*) *infantum* amplicons showing a single peak (unpaired t-test with Welch’s correction, *t* = 8.77, *P* ˂ 0.001) (Table 2). However, the T_m_ value distribution overlapped (Fig. 3), therefore samples having T_m_ values ˂ 84.4 °C appeared in a range of uncertainty, as well as samples undetermined due to lack of concordance between replicates or due to late amplification (i.e. C_q_ ˃ 30). Taken together, HRM analysis did not allow discrimination between *L.* (*L.*) *amazonensis* and *L.* (*L.*) *infantum* in 53.4% of cases. Therefore, an approach based on a second qPCR was needed.

**Table 2:** Tm comparison of *L.* (*L.*) *amazonensis* and *L.* (*L.*) *infantum* amplicons showing a single HRM peak

	Number of replicates	T_m_ Mean *±* SD (SE)	D’Agostino & Pearson omnibus normality test		Unpaired *t*-test with Welch’s correction
			K2	*P*-value	*P*-value
*L.* (*L*.) *amazonensis*	16	83.91 *±* 0.20 (0.05)	0.1456	0.93	< 0.001
*L.* (*L*.) *infantum*	114	84.44 *±* 0.36 (0.03)	1.28	0.53	

*Abbreviations*: *T*_*m*_ high resolution melting temperature, *SD* standard deviation, *SE* standard error

### qPCR assay for amplification of *L.* (*L.*) *amazonensis*

A qPCR assay which amplified an 88 bp sequence from *L.* (*L.*) *amazonensis* (qPCR-ama) was designed using a new forward primer (LMi-amaF) and the reverse primer MLR. The sequence of the LMi-amaF primer was first evaluated *in silico* using BLAST. We found 100% identity in 10 out of 16 (62.5%) sequences of *L.* (*L.*) *amazonensis* kDNA minicircle present in GenBank (Additional file 2: Figure S2). Notably, no sequence of *L.* (*L.*) *infantum* matched with the LMi-amaF primer. Moreover, only one sequence out of 101 (1%) of *L.* (*L.*) *donovani*, 3 out of 11 (27%) of *L.* (*L.*) *Mexicana*, and 7 out of 24 (29%) of *L.* (*V.*) *braziliensis* matched with LMi-amaF sequence, accounting for the largest presence of the minicircle subclass matching LMi-amaF primer in *L.* (*L.*) *amazonensis*.

**Fig. 4:**
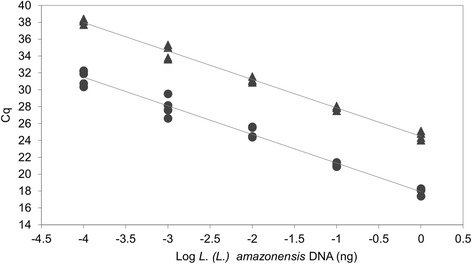
qPCR-ama standard curves constructed with serial dilutions of *L*. (*L*.) *amazonensis* MHOM/BR/00/LTB0016 DNA. The standard curves were obtained with serial dilutions ranging from 1.0 to 0.0001 ng/tube, spiked (upper curve, y = -3.3720x + 24.4850; *R* ^2^ = 0.9915) or non-spiked (lower curve, y =-3.3933x + 17.9160; *R* ^2^ = 0.9803) with 30 ng human DNA. Each point derived from a duplicate of two independent experiments

The efficiency and detection limit of the qPCR-ama assay were evaluated using 10-fold serial dilutions of *L.* (*L.*) *amazonensis* MHOM/BR/00/LTB0016 DNA (from 1.0 to 1 × 10^-4^ ng) in 2 independent experiments in duplicate or triplicate. There was a linear correlation between the log of DNA concentration and C_q_ value (slope =-3.39, *R* ^2^ = 0.98) with a reaction efficiency of 97% (Fig. 4). To verify the interference of host DNA, the standard curve was also spiked with 30 ng of purified human DNA. The presence of host DNA delayed the C_q_ values in the standard curve, while the assay efficiency remained similar (Fig. 4). The detection limit was 0.1 pg of parasite DNA in the spiked samples, corresponding roughly to 1.3 parasites/PCR tube. This value was determined by the genome size of *L.* (*L.*) *amazonensis* (29.6 Mb, 64.8 fg for the diploid genome) [25], adding 9.7 fg (15%) kDNA [26]. The calculated total DNA for a single parasite was 74.5 fg.

**Fig. 5:**
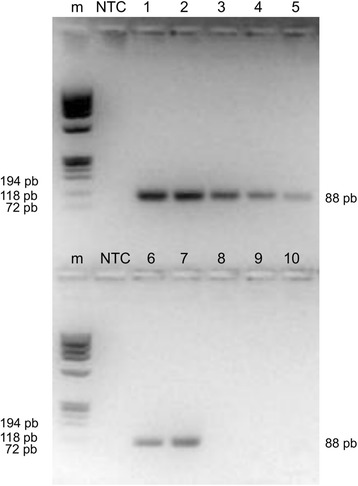
Electrophoretic analysis of qPCR-ama products. Lanes 1–5: *L.* (*L.*) *amazonensis* MHOM/BR/00/LTB0016 serial dilution ranging from 1.0 to 0.0001 ng DNA/reaction tube; Lane 6: *L.* (*L.*) *amazonensis* isolate; Lane 7: *L.* (*L.*) *amazonensis* IFLA/BR/67/PH8; Lane 8: *Trypanosoma cruzi*; Lane 9: human DNA; Lane 10 canine DNA. *Abbreviations*: m, marker 9; NTC, no template control

The specificity of the primers was first tested using two strains and one isolate of *L.* (*L.*) *amazonensis*, *Trypanosoma cruzi* and host DNA (human or canine). No amplification was observed when using *T. cruzi*, human or canine DNA, while all *L.* (*L.*) *amazonensis* strains were positive on qPCR (Fig. 5). C_q_ values were much lower compared to C_q_ obtained with qPCR-ML using the same template amount (Table 3). The qPCR-ama was then tested with strains of *L.* (*V.*) *guyaniensis, L.* (*V.*) *panamensis, L.* (*V.*) *braziliensis* and *L.* (*L.*) *infantum.* The results showed that *L.* (*V.*) *guyaniensis, L.* (*V.*) *panamensis*, *L.* (*V.*) *braziliensis* also amplified. However, these species could be distinguished from *Leishmania* (*L.*) species by qPCR-ML and HRM analysis. DNA from *L.* (*L.*) *infantum* isolates/strains did amplify, but C_q_ values were dramatically increased compared to qPCR-ML. If template DNA was diluted to have C_q_ ˃ 25 in qPCR-ML, there was no detectable amplification from qPCR-ama (Table 3). As evidence of this, the canine clinical samples tested with qPCR-ML (with C_q_ ˃ 25) had negative results with qPCR-ama, confirming infection by *L.* (*L.*) *infantum* (Table 4). Combined evaluation of qPCR-ML and qPCR-ama C_q_ values provided discrimination between *L.* (*L.*) *infantum* and *L.* (*L.*) *amazonensis* in all tested samples.

**Table 3:** qPCR-ML and qPCR-ama results

Species	qPCR ML (C_q_ ± SD)	qPCR-ama (C_q_ ± SD)				
	Undiluted	Undiluted	1:10	1:100	1:1,000	1:10,000
*L.* (*L.*) *infantum* MHOM/TN/80/IPT1	17.26 ± 0.19	negative	negative	negative	nt	nt
*L.* (*L.*) *infantum* MHOM/FR/78/LEM75	15.30 ± 0.41	nt	33.80 ± 0.33	37.92 ± 2.23	negative	negative
*L.* (*L.*) *infantum* MHOM/ES/81/BCN1	15.80 ± 0.77	33.70 ± 0.33	38.01 ± 1.36	negative	negative	nt
*L.* (*L.*) *infantum* MHOM/DZ/82/LIPA59	16.43 ± 0.47	33.03 ± 0.61	nt	nt	negative	negative
*L.* (*L.*) *infantum* MHOM/IT/93/ISS822	14.73 ± 0.42	33.89 ± 0.66	nt	nt	negative	negative
*L.* (*L.*) *infantum* MHOM/IT/86/ISS218	15.42 ± 1.03	32.44 ± 1.18	nt	nt	negative	negative
*L.* (*L.*) *infantum* Clinical isolate V2921	17.09 ± 0.12	29.08 ± 0.13	nt	nt	negative^a^	negative
*L.* (*L.*) *infantum* Clinical isolate 31u	14.30 ± 0.27	28.73 ± 0.78	nt	nt	negative^a^	negative
*L.* (*L.*) *infantum* Clinical isolate 49u	15.71 ± 0.78	30.02 ± 0.01	nt	nt	negative	negative
*L.* (*L.*) *infantum* Clinical isolate 791	14.88 ± 0.51	27.76 ± 0.07	nt	nt	38.81 ± 0.93	negative
*L.* (*L.*) *infantum* Clinical isolate 10816	13.53 ± 0.46	26.53 ± 0.11	nt	nt	36.67 ± 2.08	negative
*L.* (*L.*) *amazonensis* MHOM/BR/00/LTB0016	23.79 ± 0.25	17.97 ± 0.40	21.08 ± 0.23	25.00 ± 0.66	27.97 ± 1.21	31.49 ± 0.88
*L.* (*L.*) *amazonensis* IFLA/BR/67/PH8	34.42 ± 0.02	15.32 ± 0.01	19.33 ± 0.32	nt	nt	nt
*L.* (*L.*) *amazonensis* Clinical isolate	31.66 ± 0.19	20.87 ± 0.24	24.12 ± 0.14	nt	nt	nt
*L.* (*V.*) *panamensis* Clinical isolate	22.96 ± 0.28	35.57 ± 0.56	nt	negative	nt	nt
*L.* (*V.*) *guyanensis* Clinical isolate	29.71 ± 0.32	33.30 ± 0.17	36.78 ± 0.46	negative	nt	nt
*L.* (*V.*) *braziliensis* Clinical isolate	28.15 ± 0.85	37.87 ± 0.86	negative	negative	nt	nt
*L.* (*V.*) *braziliensis* MHOM/BR/75/M2904	26.07 ± 1.80	38.19 ± 0.04	negative	negative	nt	nt

*Abbreviation*: *nt* not tested

^a^One replicate negative

**Table 4:** qPCR-ML and qPCR-ama results in canine clinical samples

Canine conjunctival swab sample	qPCR-ML (C_q_ ± SD)	qPCR-ama (C_q_ ± SD)
1	29.15 ± 0.84	negative
2	31.93 ± 1.13	negative
3	31.49 ± 0.69	negative
4	34.06 ± 0.76	negative
5	33.61 ± 0.18	negative
6	27.32 ± 0.96	negative

## Discussion

Previous work has shown that the *Leishmania* (*Leishmania*) and *Leishmania* (*Viannia*) subgenera could be distinguished by exploiting kDNA variability through HRM analysis of qPCR-ML amplicons [13]. Here, several qPCR-ML amplicons from different *Leishmania* species were sequenced, indicating the presence of different subclasses of minicircles amplified from the same species. Notably, the presence of over 100 minicircles classes encoding different gRNAs in a strain of *L. tarentolae* has been reported and quantified using NGS technology, indicating the heterogeneity of the mitochondrial genomes in this parasite [27]. The *EcoR*I partial digestion of the amplicon obtained from *L.* (*L.*) *amazonensis* isolate confirmed the presence of different sequences in the same PCR product. In fact, the sequence of the non-digested product evidenced a subclass of molecules without the *EcoR*I site, clustering with *L.* (*L.*) *infantum* sequences. The fact that the sequence of this product was not visible in the first sequence analysis can be explained by a relatively low presence of this minicircle subclass.

*Leishmania* (*L.*) *infantum* and *L.* (*L.*) *amazonensis* infect both humans and dogs, and they can coexist in the same geographical area; therefore, their differentiation is critical for correct treatment. Notably, Sanches et al. [28] showed a high percentage of *L.* (*L.*) *amazonesis* infection in naturally infected dogs in an endemic area, underlining the necessity to discriminate these two species not only in human but also in veterinary medicine. Since HRM analysis resulted inconclusive for discrimination of *L.* (*L.*) *infantum* and *L.* (*L.*) *amazonensis* in about 53% of samples, a new SYBR-green qPCR assay (qPCR-ama) was designed to amplify a minicircle subclass preponderant in *L.* (*L.*) *amazonensis,* rather than targeting a hypothetical species-specific sequence. In fact, several PCR assays designed on minicircles also reported amplification of non-intended species [17, 29]. Moreover, Gomes et al. [30] showed that non-target organisms such as *T. cruzi* could be amplified, even if C_q_ values are ˃ 30. Therefore, the performance of different qPCR assays in series or the standardisation of multiplex qPCR appeared to be the only way of properly identifying different *Leishmania* species [31]. Due to the difficulties in designing species-specific primers, we based our qPCR assay on the evaluation of relative abundance of minicircle subclasses. We observed that qPCR-ML amplified several *Leishmania* species including *L.* (*L.*) *amazonensis* but to a much less sensitivity (higher C_q_). The opposite was found for qPCR-ama: in this case, *L.* (*L.*) *amazonensis* was amplified much more efficiently (lower C_q_) compared to the other tested species, because primers were designed on a minicircle sequence that was more abundant in this species. Therefore, we have been focusing on C_q_ rather than presence or absence of an amplification curve. Hence, a minicircle subclass most represented in *L.* (*L.*) *amazonensis* was used as a target to differentiate *L*. (*L*.) *amazonensis* and *L.* (*L*.) *infantum*. The template DNA can be diluted to make undetectable the low-represented minicircle subclasses*.* The data in Table 3 show clearly this point: if DNA template from *L.* (*L.*) *infantum* was diluted to have C_q_ ˃ 25 in qPCR-ML (considering 1:10 dilution equivalent to ~ 3.3 cycles increase), the qPCR-ama turned negative. On the contrary, under appropriate dilution conditions, the qPCR-ama will give positive amplification only for *L.* (*L.*) *amazonensis* species (Table 3). These conditions could also be met by clinical samples, in which pathogen DNA is low represented respect to host DNA and the C_q_ can be delayed (as evidenced in the qPCR-ama standard curves).

The results of *in silico* analysis accounted for the possibility to amplify more *L.* (*L.*) *amazonensis* strains other those tested in this work, despite the database does not give information about the relative abundance of the kDNA minicircle sequences into the different strains. For example, the kDNA minicircle sequence of strain IFLA/BR/67/PH8 (GenBank M21325) shows a mismatch in the LMi-amaF sequence (Additional file 2: Figure S2) but it was efficiently amplified in our assay (Table 3), probably due to the presence of another minicircle subclass matching the primer sequence.

**Fig. 6:**
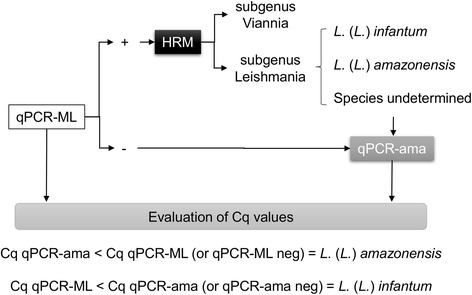
A possible diagnostic approach based on qPCR and HRM analysis. The qPCR-ML followed by HRM analysis is performed. In the case of lack of amplification, or if HRM results are inconclusive, the qPCR-ama can also be performed. The evaluation of C_q_ values for both assays will allow discriminating between *L.* (*L.*) *infantum* and *L.* (*L.*) *amazonensis*

Taken together, a new diagnostic approach for *Leishmania* species discrimination from clinical samples or isolates has been developed: first, a qPCR-ML followed by HRM analysis is performed. In the case of lack of amplification, or if HRM results are inconclusive, the qPCR-ama assay is performed, and the evaluation of C_q_ values for both assays will allow the discrimination of the two species (Fig. 6). This approach is affordable for any molecular diagnostic laboratory equipped with an HRM qPCR instrument since it is based on an SYBR green chemistry and requires only three primers. Moreover, since the cost per reaction is relatively low (less than 1 €) and the PCR conditions are the same, the two assays could be run simultaneously to speed up the diagnostic process.

## Conclusions

The new approach to differentiate *L.* (*L.*) *amazonensis* and *L.* (*L.*) *infantum* infections in geographical regions in which these species co-exist could be a useful diagnosis tool. Due to qPCR sensitivity, this approach will allow species differentiation from isolated parasites and from DNA extracted from clinical samples. This study represents a proof of concept: more samples should be analysed, and accuracy studies should be performed in clinical environments to validate the real utility of this method. Moreover, more species could be tested to extend the feasibility of this approach to other species or complex.

## Additional files

Additional file 1: Figure S1. RFLP analysis. Amplicons obtained with qPCR-ML were digested with *EcoR*I as described in methods. Digestion mixtures were analysed on a 3% high-resolution MetaPhor gel. Only the amplicon from *L.* (*L.*) *amazonensis* isolate was partially restricted in fragments of 64 and 48 bp. 1) *L.* (*L.*) *infantum* MHOM/TN/80/IPT1; 2) *L.* (*L.*) *amazonensis* isolate; 3) *L.* (*V.*) *guyanensis* isolate; 4) *L.* (*V.*) *panamensis* isolate; 5) *L.* (*V.*) *braziliensis* isolate; 6) canine clinical sample A sx; m: marker 9. (PPTX 47 kb)

Additional file 2: Figure S2. CLUSTAL multiple alignments of 16 *L.* (*L.*) *amazonensis* kDNA minicircle sequences retrieved from Genbank (partial sequences). The alignment was performed by MUSCLE with default options. The boxes indicate the positions of primers LMi-amaF and LMR; the sequences perfectly matching the LMi-amaF primer are highlighted. (DOCX 16 kb)

## Abbreviations

BC: Buffy coat
CL: Cutaneous leishmaniasis
C_q_: Threshold cycle
CS: Conjunctival swab
HRM: High-resolution melt
kDNA: Kinetoplast DNA
qPCR: Quantitative real-time PCR
VL: Visceral leishmaniasis
