# Supplementary figures and images for: The use of kDNA minicircle subclass relative abundance to differentiate between Leishmania (L.) infantum and Leishmania (L.) amazonensis
Source: Parasit Vectors. 2017 May 16;10:239. doi: 10.1186/s13071-017-2181-x (PMC5434583; doi:10.1186/s13071-017-2181-x)

## Slide 1
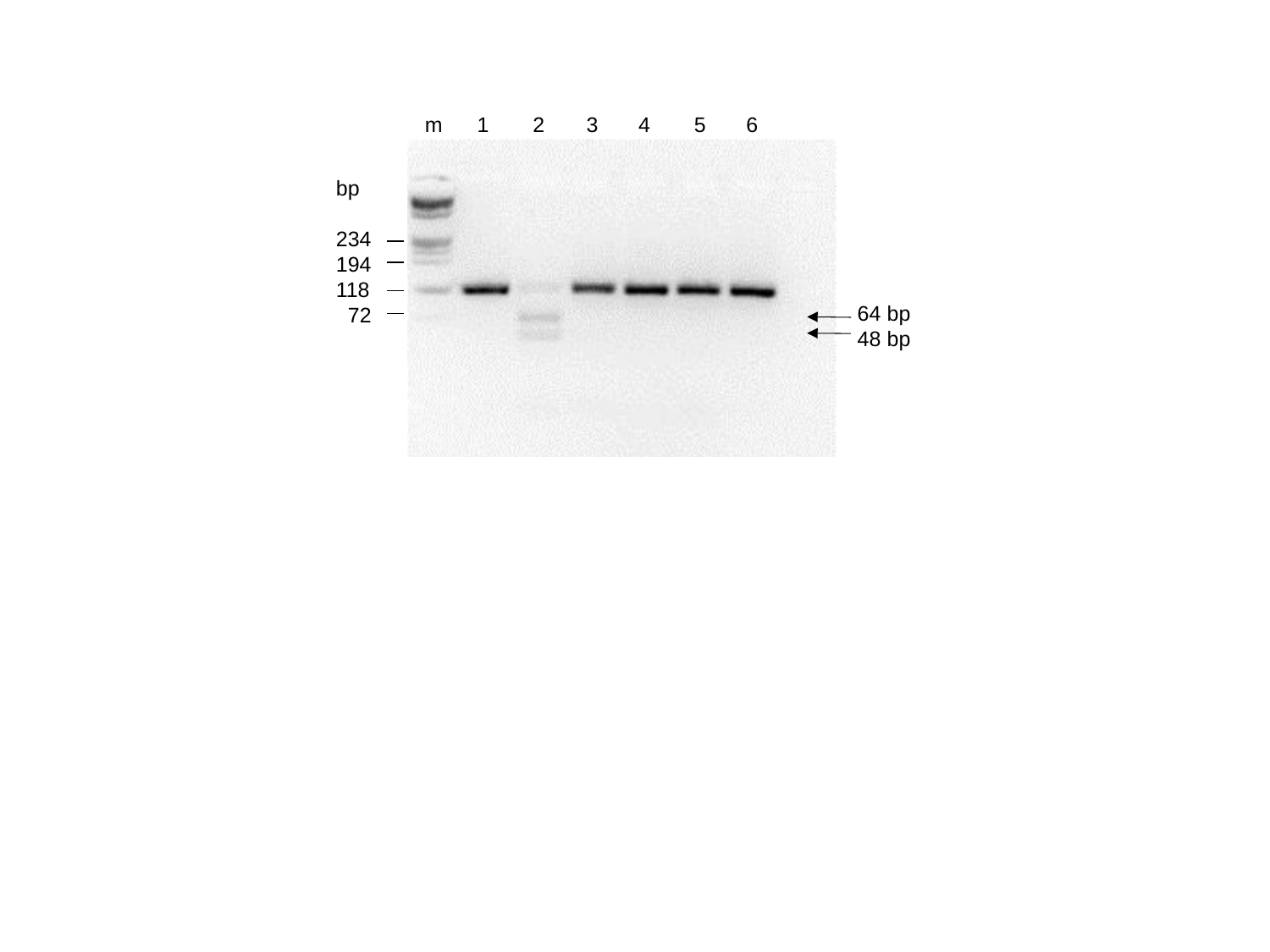

m
1
2
3
4
5
6
bp
234
194
118
64 bp
48 bp
 72

Supplement: Supplementary file 1 — RFLP analysis. Amplicons obtained with qPCR-ML were digested with EcoRI as described in methods. Digestion mixtures were analysed on a 3% high-resolution MetaPhor gel. Only the amplicon from L. (L.) amazonensis isolate was partially restricted in fragments of 64 and 48 bp. 1) L. (L.) infantum MHOM/TN/80/IPT1; 2) L. (L.) amazonensis isolate; 3) L. (V.) guyanensis isolate; 4) L. (V.) panamensis isolate; 5) L. (V.) braziliensis isolate; 6) canine clinical sample A sx; m: marker 9. (PPTX 47 kb) [file 13071_2017_2181_MOESM1_ESM.pptx]
