# Supplementary material for: The use of kDNA minicircle subclass relative abundance to differentiate between Leishmania (L.) infantum and Leishmania (L.) amazonensis
Source: Parasit Vectors. 2017 May 16;10:239. doi: 10.1186/s13071-017-2181-x (PMC5434583; doi:10.1186/s13071-017-2181-x)
Supplement: Supplementary file 2 — CLUSTAL multiple alignments of 16 L. (L.) amazonensis kDNA minicircle sequences retrieved from Genbank (partial sequences). The alignment was performed by MUSCLE with default options. The boxes indicate the positions of primers LMi-amaF and LMR; the sequences perfectly matching the LMi-amaF primer are highlighted. (DOCX 16 kb) [file 13071_2017_2181_MOESM2_ESM.docx]

M94088.1 GGGGCGTTCTGCGAATTTCG-AAAAATCGATACAGAAACCCCGTTCAAAAATTCCAGGGA

M94089.1 GGGGCGTTCTGCGAATTTCG-AAAAATCGATACAGAAACCCCGTTC-AAAAATCCACGGA

U19810.1 GGGGCGTTCTGCGGATTCGGGAAAAATGAGTGCGAAACCCCGGTTC-ATAATTTGGGGGA

EU370873.1 GGGGCGTTCTGCGAAAACCGGAAAATTGATAGCAGAAACCCCGTTC-ATAATTTGGGGGA

M94091.1 GGGGCGTTCTGCGAATTTGGGAAAAATGAGTGCAGAAACCCCGTTC-ATATTTTGGGGAA

EU370871.1 GGGGCGTTCTGCGAAA-CGGGGAAAATGAGTGCAGAAACCCCGTTC-ATATTTTGGGGAA

M21327.1 GGGGCGTTCTGCGGAAATGGCAAAAATGAGTGCAGAAACCCCGTTC-ATATTTTGGGGGA

EU370872.1 GGGGCGTTCTGCGAAATCGGCAAAAATGAGTGCAGAAACCCCGTTC-ATATTTTTGGGGA

M21326.1 GGGGCGTTCTGCGGAAACCTCAAAAATGAGTGCAGAAACCCCGTTC-ATATTTTGGGGGA

M21325.1 GGGGCGTTCT--CAAAATGGCAAAAATGGGTGCAGAAACCCCG-TC-ATATTTTCGGGGA

EU429383.1 GGGGCGTTCTGCGGATTGGCGAAAAATGAGTGCAGAAACCCCGTTC-ATATTTTGGGCAA

M94090.1 GGGGCGTTCTGCGAAAATGGCAAAAATGAGTGCAGAAACCCCGTTC-ATAATTTAGGG--

EU370874.1 GGGGCGTTCTGCGAAACCGG-AAAAATGAGTGCAGAAACCCCGTTC-ATAATTTCTCCGA

EU370870.1 GGGGCGTTCTGCGAAAACCGGAAAAATGGGTGCAGAAACCCCGTTC-ATATTTTGGCCAA

EU370875.1 GGGGCGTTCTGCGAAAATGGGAAAAATGAGTGCAGAAACCCCGTTC-ATAATTTGGCCAA

EU370869.1 GGGGCGTTCTGCGAAAATGGCAAAAATGAGTGCAGAAACCCCGTTC-ATATTTTGACCAA

********** * *** * * ** *** * ** * * *

M94088.1 AAATGCCATTTTTGGCCTTGGGGCGTGCAAACTGGGGGTTGGTGTAAAATAGGCCGGGTG

M94089.1 AAATGCCATTTTTGGCCTCGGGGCGTGCAAACTGGGGGTTGGTGTAAAATAGGCCGGGTG

U19810.1 TTTTGGAGAATTCCGGCTCCGAGGCTCGAAACTGGGGGTTGGTGTAAAATAGGGGCGGCT

EU370873.1 TTTCCGGGAATTCCGGCTCCGGGGCGCGAAACTGGGGGTTGGTGTAAAATAGG-------

M94091.1 TTTTG----------------------------------------------GG-------

EU370871.1 TTTTGGCCGAAAATGCCTCGGGCGGTGAAAACTGGGGGTTGGTGTAAAATAGG-------

M21327.1 ATTCCTCGATTTTCGGCTCGGGGGGCTGAAACTGGGGGTTGGTGTAAAATAGGGGCGGCT

EU370872.1 ATTCGAGGAATTTCGGCTCCGGCGGTGAAAACTGGGGGTTGGTGTAAAATAGG-------

M21326.1 TTTTTGGGAATTTCGGTTCGGACGGTGGAAACTGGGGGTTGGTGTAAAATAGGGGCGGCT

M21325.1 ATTCGGGGAATTTCGGCTCGGGCGGTGAAAACTGGGGGTTGGTGTAAAATAGGGGCGGCT

EU429383.1 AAATCCCGAAATTCGGCTCGGACGGTCACAACTGGGGGTTGGTGTAAAATAGGGGCGGCT

M94090.1 ------------------------------------------------------------

EU370874.1 AAATCCCGAAATTCGGCTCGGACGGTCACAACTGGGGGTTGGTGTAAAATAGG-------

EU370870.1 AAATCTCGAAATTCGGCTCGGACGGTCACAACTGGGGGTTGGTGTAAAATAGG-------

EU370875.1 AAATCCTGGAAATCGGCTCGGGCGGTCACAACTGGGGGTTGGTGTAAAATAGG-------

EU370869.1 AAATCCCGAAATTCGGCTCGGGCGGTCACAACTGGGGGTTGGTGTAAAATAGG-------
